# A novel high-yield synthesis of aminoacyl *p*-nitroanilines and aminoacyl 7-amino-4-methylcoumarins: Important synthons for the synthesis of chromogenic/fluorogenic protease substrates

**DOI:** 10.3762/bjoc.7.117

**Published:** 2011-07-27

**Authors:** Xinghua Wu, Yu Chen, Herve Aloysius, Longqin Hu

**Affiliations:** 1 Department of Medicinal Chemistry, Ernest Mario School of Pharmacy, Rutgers, The State University of New Jersey, Piscataway, New Jersey 08854; 2 The Cancer Institute of New Jersey, New Brunswick, New Jersey 08901

**Keywords:** 7-amino-4-methylcoumarin, *p*-nitroaniline, proteolytic substrate, selenocarboxylate/azide amidation, synthon

## Abstract

Aminoacyl *p*-nitroaniline (aminoacyl-*p*NA) and aminoacyl 7-amino-4-methylcoumarin (aminoacyl-AMC) are important synthons for the synthesis of chromogenic/fluorogenic protease substrates. A new efficient method was developed to synthesize aminoacyl-*p*NA and aminoacyl-AMC derivatives in excellent yields starting from either amino acids or their corresponding commercially available *N*-hydroxysuccinimide esters. The method involved the in situ formation of selenocarboxylate intermediate of protected amino acids and the subsequent non-nucleophilic amidation with an azide. Common protecting groups used in amino acid/peptide chemistry were all well-tolerated. The method was also successfully applied to the synthesis of a dipeptide conjugate, indicating that the methodology is applicable to the synthesis of chromogenic substrates containing short peptides. The method has general applicability to the synthesis of chromogenic and fluorogenic peptide substrates and represents a convenient and high-yield synthesis of *N*^α^-protected-aminoacyl-*p*NAs/AMCs, providing easy access to these important synthons for the construction of chromogenic/fluorogenic protease substrates through fragment condensation or stepwise elongation.

## Introduction

Chromogenic and fluorogenic amino acid/peptide conjugates are widely used as substrates in enzyme assays for protease activity and specificity [1]. Proteolytic cleavage of the amino acid/peptide conjugates liberates the free chromophore or fluorophore, allowing the convenient determination of the rate of enzyme catalysis with a UV or fluorescence spectrophotometer. *p*-Nitroaniline (*p*NA) is one of the most commonly used chromogenic reagents. The synthesis of peptide-*p*NAs usually involves formation of aminoacyl-*p*NAs through the attachment of P1 amino acid to the amino group of *p*-nitroaniline, followed by fragment condensation or stepwise elongation [P1 amino acid refers to the residue immediately amino terminal to the scissile bond in a proteinase substrate]. However, the synthesis of aminoacyl-*p*NAs is particularly problematic due to the poor nucleophilicity of the aromatic amino group, which is further deactivated by the electron-withdrawing nitro group at the *para* position. Direct acylation of *p*-nitroaniline using common coupling methods, such as DCC [2], HOBt activated ester [3], acyl chloride [4], and mixed anhydride methods [5–8], did not afford satisfactory yields. A phosphorus trichloride method was developed to improve the acylation yield through a phospho–azo mechanism [9]. However, this method is only applicable to amino acids protected with acid resistant groups such as Cbz and Pht. The use of phosphorus pentaoxide for peptide synthesis was also reported, but racemization could occur under these reaction conditions [10–11]. Phosphorus oxychloride was another condensing agent used to synthesize various *N*-protected aminoacyl-*p*NAs in yields between 70% and 90% [12]. The proposed mechanism implies in situ activation of carboxylic acid by formation of the active mixed anhydride with phosphorodichloridic acid. Although the authors demonstrated that the synthesis was simple and free of racemization, this method required unfavorable anhydrous pyridine as a solvent to suppress the potential removal of the Boc protecting group of amino acids. To increase nucleophilicity of the aromatic amino group and hence improve the acylation yield, *p*-(Boc-amino)aniline was used in place of *p*-nitroaniline as the starting material for amidation, followed by selective removal of the Boc protecting group and oxidation of the free amino group to the nitro group [13]. This method, however, is not applicable to methionine, tyrosine, tryptophan and cysteine, all of which are sensitive to the oxidation conditions used. As an alternative, *N*^α^-protected amino acids were used to condense with *p*-nitrophenyl isocyanate to form the *N*^α^-protected aminoacyl-*p*NAs [14]. However, commercially available *p*-nitrophenyl isocyanate often contains impurities due to degradation during storage; these impurities must be removed before use. In addition, a significant amount of hydantoin by-product was found in the reaction mixture. Although the addition of DMAP improved the amidation yields, the formation of hydantoin by-product also increased [15]. A modified procedure using Curtius rearrangement with diphenyl phosphorazidate (DPPA) was reported to minimize the side reaction, affording moderate to excellent yields (54–95%) of the desired *N*^α^-protected aminoacyl-*p*NAs [16]. However, only Boc protected amino acids were used in the reported study, with three examples provided. The enzymatic synthesis of aminoacyl-*p*NAs was also reported recently [17]. Although this method is free of racemization, it does not result in satisfactory yields. Because of these limitations of the existing methods, there is still a need to develop a generally applicable method for the synthesis of aminoacyl-*p*NAs. Conjugation of *N*^α^-protected amino acids with fluorogenic 7-amino-4-methylcoumarin (AMC) is similarly as problematic as *p*-nitroaniline due to the low nucleophilicity of the 7-aromatic amino group. Thus, the method developed for the synthesis of aminoacyl-*p*NAs could also be applied to the synthesis of aminoacyl-AMC derivatives.

We previously described a novel selenocarboxylate/azide amidation reaction [18–19] and its application to directly couple amino acids with azides [20]. We demonstrated that our selenocarboxylate/azide amidation does not involve reduction of the azide to amine and subsequent standard coupling reaction [18–19]. We also demonstrated that the selenocarboxylate/azide amidation was highly chemoselective, mild, and free of racemization in the coupling step [20]; was compatible with common protecting groups used in peptide chemistry including Fmoc, Boc, Cbz, and Trt; and more importantly, worked very efficiently for electron-deficient aromatic azides substituted with an electron-withdrawing group [18–20]. We postulated that the selenocarboxylate/azide amidation could be a good solution to the problem of synthesizing aminoacyl-*p*NAs and aminoacyl-AMCs. Instead of *p*-nitroaniline and 7-amino-4-methylcoumarin, *p*-nitrophenyl azide and 7-azido-4-methylcoumarin would be used to react with the selenocarboxylates derived from *N*^α^-protected amino acids to form the desired amides. The azides can be readily prepared in excellent yields through diazotization of *p*-nitroaniline and 7-amino-4-methylcoumarin, followed by treatment with sodium azide, and, more importantly, the azides are stable and can be stored for a long period of time without significant degradation. More recently, we published a facile procedure for the preparation of *N*^α^-protected amino selenocarboxylate that could be easily used in situ [21]. Herein, we report a practical method for the preparation of aminoacyl-*p*NA and aminoacyl-AMC derivatives through the selenocarboxylate/azide amidation strategy, under aqueous or alcoholic reaction conditions, with excellent yields.

## Results and Discussion

An efficient method was developed to synthesize aminoacyl-*p*NAs and aminoacyl-AMCs in excellent yields as shown in Table 1 and Table 2, through the reaction of *N*-hydroxysuccinimide (HOSu)-activated esters or mixed anhydrides of *N*^α^-protected amino acids with sodium hydrogen selenide to form their corresponding *N*^α^-protected amino selenocarboxylate, followed by the coupling of the selenocarboxylate with *p*-nitrophenyl azide and 7-azido-4-methylcoumarin. When *N*^α^-protected amino acid-OSu activated ester is not commercially available, the mixed anhydrides of *N*^α^-protected amino acids were prepared from *N*^α^-protected amino acids in situ, prior to use. *N*^α^-Protected amino acid-OSu esters or mixed anhydrides were mixed with a stoichiometric amount of freshly prepared NaHSe [22] to generate the corresponding selenocarboxylates in situ, which were then reacted with azides to form the corresponding *N*^α^-protected aminoacyl-*p*NAs or *N*^α^-protected aminoacyl-AMCs. The amount of amino selenocarboxylates generated was slightly in excess (1.2 equiv) relative to the azide used. The reaction was typically complete within 0.5 h at 0 °C for OSu-activated esters or −10 °C for mixed anhydrides. For sterically hindered amino acid OSu esters, such as isoleucine, valine and threonine, however, the reaction was slower and required room temperature to reach completion. After the completion of selenocarboxylation as monitored by LC-MS, a solution of azide in THF was added by syringe or cannulation. The reaction started immediately with the precipitation of gray selenium and the formation of nitrogen gas, and was complete within 2 h at room temperature. Although it was difficult to characterize the selenocarboxylate intermediates using NMR due to their short half-life and their sensitivity to ambient oxygen, we were able to monitor the formation of *N*^α^-protected amino selenocarboxylates through their deprotonated molecular ions and their characteristic Se isotopic patterns with LC-MS in ES^−^ mode as previously reported [21].

As shown in Table 1, Cbz, Boc, Fmoc and Trt protecting groups were all well tolerated under our conditions, with OSu-activated esters of *N*^α^-protected amino acids as the starting material, and excellent yields of aminoacyl-*p*NAs were obtained. We also found that the hydroxy group of tyrosine and threonine did not need to be protected. No significant intermolecular esterification was observed under the present reaction conditions and the desired *p*-nitroanilides were obtained in excellent yields (Table 1, entries 2 and 3). Furthermore, we prepared an aqueous solution of NaHSe and treated it with Cbz-Gly-OSu in 50% aqueous THF to produce Cbz-Gly-SeNa. The subsequent amidation with *p*-nitrophenyl azide afforded the desired Cbz-Gly-*p*NA (**1**) in 95% yield (Table 1, entry 1), indicating that the reaction could be carried out under aqueous conditions. When Fmoc-Ile-OSu reacted with NaHSe, a higher reaction temperature (room temperature) was required to complete the selenocarboxylation, suggesting the presence of steric hindrance. However, once the Fmoc-Ile-SeNa was formed, it quickly reacted with *p*-nitrophenyl azide to provide Fmoc-Ile-*p*NA (**4**) in an excellent yield. This indicated that the steric hindrance of selenocarboxylates did not play a significant role in the amidation step (Table 1, entry 4), which is consistent with our previous observation that the rate of selenocarboxylate/azide amidation primarily depended on the electronic properties of the azide [20]. Furthermore, the indole unit of tryptophan did not need protection and was well-tolerated without affecting the yield of the amidation reaction (Table 1, entry 7). This newly developed selenocarboxylate/azide amidation strategy also provided easy access to aminoacyl-AMC conjugates. 7-Azido-4-methylcoumarin was readily prepared from 7-AMC by the standard diazotization/sodium azide protocol in a yield of 92%. Under the same reaction conditions described above, the amidation of 7-azido-4-methylcoumarin with various amino selenocarboxylates gave the desired *N*^α^-protected aminoacyl-AMCs in excellent yields (82–92%) (Table 1, entries 9 to 14). The yields were slightly lower than the yields of the corresponding *p*-nitroanilides prepared under the same conditions. This is consistent with the previously observed trend that azides that were more electron-deficient gave better yields.

**Table 1:** Synthesis of *N^α^*-protected aminoacyl-*p*NAs and aminoacyl-AMCs through selenocarboxylation of *N^α^*-protected amino acid OSu esters with NaHSe, followed by reaction with the azides (Procedure A).

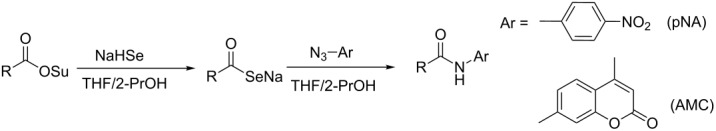			

Entry	Amino acid-OSu ester	Product	Yield (%)^a^

1	Cbz-Gly-OSu	Cbz-Gly-*p*NA (**1**)	98 (95^b^)
2	Cbz-Tyr-OSu	Cbz-Tyr-*p*NA (**2**)	90 (88^b^)
3	Fmoc-Thr-OSu	Fmoc-Thr-*p*NA (**3**)	91^c^
4	Fmoc-Ile-OSu	Fmoc-Ile-*p*NA (**4**)	90^c^
5	Boc-Phe-OSu	Boc-Phe-*p*NA (**5**)	91
6	Fmoc-Met-OSu	Fmoc-Met-*p*NA (**6**)	92
7	Fmoc-Trp-OSu	Fmoc-Trp-*p*NA (**7**)	94
8	Fmoc-His(Trt)-OSu	Fmoc-His(Trt)-*p*NA (**8**)	91
9	Cbz-Gly-OSu	Cbz-Gly-AMC (**9**)	92 (89^b^)
10	Cbz-Tyr-OSu	Cbz-Tyr-AMC (**10**)	86 (84^b^)
11	Boc-Phe-OSu	Boc-Phe-AMC (**11**)	86
12	Fmoc-Met-OSu	Fmoc-Met-AMC (**12**)	82
13	Fmoc-Trp-OSu	Fmoc-Trp-AMC (**13**)	82
14	Fmoc-His(Trt)-OSu	Fmoc-His(Trt)-AMC (**14**)	82

Conditions: ^a^NaHSe (1.2 equiv) and Nα-protected amino acid-OSu (1.2 equiv) in THF/2-PrOH at 0 °C, then the azide (1.0 equiv); ^b^NaHSe (1.2 equiv) and amino acid OSu (1.2 equiv) in THF/H2O at 0 °C, then the azide (1.0 equiv); ^c^NaHSe (1.2 equiv) and *N^α^*-protected amino acid-OSu (1.2 equiv) in THF/2-PrOH at r.t., then the azide (1.0 equiv).

For amino acids whose OSu-activated esters are not commercially available or difficult to prepare, we developed a 3-step one-pot procedure (Procedure B) starting from *N*^α^-protected amino acids: 1) The amino acid was first activated to form the corresponding mixed anhydride; 2) the resulting mixed anhydride was converted in situ to the selenocarboxylate by reaction with NaHSe; and 3) the in situ generated selenocarboxylate reacted immediately with the azide. Isopropyl chloroformate was chosen to activate amino acids because the corresponding mixed anhydrides were relatively more resistant to hydrolysis or alcoholysis. As shown in Table 2, Boc-Gln-*p*NA (**15**) and Cbz-Ser-*p*NA (**17**) were successfully synthesized in excellent yields of 91% and 86%, respectively, without the need to protect the side chain functional groups (Table 2, entries 1 and 3). *N*^α^-protected arginine *p*NAs are of special interest since they are often the starting materials for the synthesis of chromogenic substrates for serine proteases. Due to the presence of the guanidino group, the synthesis of *N*^α^-protected Arg-*p*NA was especially challenging. We used Boc-Arg-OH∙HCl directly without protecting the guanidino group and found that Boc-Arg-OH∙HCl could be activated with isopropyl chloroformate and the activated form was converted smoothly to the corresponding selenocarboxylate followed by the reaction with *p*-nitrophenyl azide to give the *p*-nitroanilide **16** in 81% yield (Table 2, entry 2). A dipeptide-*p*NA, Boc-Ser-Phe-*p*NA (**18**), was also prepared in an excellent yield of 89% in the same manner, indicating a potential use of this methodology in the synthesis of chromogenic substrates with short peptides. The same procedure was applied to synthesize aminoacyl-AMC conjugates **19**, **20** and **11** starting from Boc-Gln-OH, Boc-Arg-OH∙HCl and Boc-Phe-OH in excellent yields (Table 2, entries 5–7). It should also be pointed out that we previously determined the extent of racemization in the one-pot 3-step selenocarboxylate/azide amidation procedure using acid hydrolysis of the product amide with 6 N HCl followed by derivatization with *o*-phthalaldehyde/*N*^α^-*tert*-butyloxycarbonyl-L-cysteine and HPLC analysis, and we concluded that the obtained amide product contained a small amount (1.7%) of the D-isomer resulting from the first step of carboxylate activation rather than the last two steps of selenocarboxylate generation and amidation [20].

**Table 2:** The 3-step one-pot selenocarboxylate/azide amidation to synthesize *N^α^*-protected aminoacyl-*p*NAs and aminoacyl-AMCs (Procedure B).

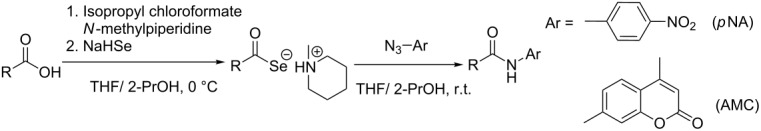			

Entry	Amino acid	Product	Yield (%)^a^

1	Boc-Gln-OH	Boc-Gln-*p*NA (**15**)	91
2	Boc-Arg-OH·HCl	Boc-Arg-*p*NA (**16**)	81
3	Cbz-Ser-OH	Cbz-Ser-*p*NA (**17**)	86
4	Boc-Ser-Phe-OH	Boc-Ser-Phe-*p*NA (**18**)	89
5	Boc-Gln-OH	Boc-Gln-AMC (**19**)	84
6	Boc-Arg-OH·HCl	Boc-Arg-AMC (**20**)	82
7	Boc-Phe-OH	Boc-Phe-AMC (**11**)	87

**Conditions**: ^a^*N*^α^-protected amino acid (1.2 equiv), isopropyl chloroformate (1.2 equiv) and *N*-methylpiperidine (1.2 equiv) in THF at −15 °C for 30 min; isopropanolic NaHSe (1.2 equiv) solution at < −10 °C for additional 30 min; then the azide (1.0 equiv), r.t., 2 h.

In summary, amino acyl conjugates of *p*NA and AMC were readily synthesized through the selenocarboxylate/azide amidation reaction. This method has many advantages, including high yields, reproducibility, short reaction time, mild reaction conditions, and compatibility with a range of organic and aqueous solvents. Most importantly, the protecting groups commonly used in amino acid and peptide chemistry, such as Fmoc, Boc, Cbz, and Trt, are all well-tolerated under the present conditions. This selenocarboxylate/azide amidation strategy represents a convenient and high-yield synthesis of *N*^α^-protected-aminoacyl-*p*NAs/AMCs, providing easy access to these important synthons for the construction of chromogenic/fluorogenic protease substrates through fragment condensation or stepwise elongation. The 3-step one pot procedure was also successfully applied to the synthesis of a dipeptide conjugate indicating that this methodology is applicable to the synthesis of chromogenic substrates containing short peptides.

## Experimental

**General methods.** Solvents were either ACS reagent grade or HPLC grade and used directly without further purification. All reaction mixtures were magnetically stirred and monitored by thin-layer chromatography (TLC) with polymer-backed F254 silica gel plates and/or LC-MS system. Flash column chromatography (FCC) was performed on a Teledyne ISCO CombiFlash Companion Automated Flash Chromatographic System with pre-packed silica gel columns. Yields were based on the chromatographically pure compounds whose structures were assigned by NMR and mass spectral data. All ^1^H and ^13^C NMR spectra were recorded on a 200 MHz Varian or a 400 MHz Bruker spectrometer at ambient temperature and calibrated using residual undeuterated solvents as the internal reference. The LC-MS monitoring was performed on a Shimadzu LCMS 2010 system with Chromolith SpeedROD RP-18e column (50 × 4.6 mm) at 1 mL/min with a 10-min gradient of 10–90% acetonitrile containing 0.1% formic acid. High resolution mass spectrometry (HRMS) was performed on a Finnagen LTQ OrbiTrap mass spectrometer.

**Preparation of an isopropanol solution of sodium hydrogenselenide** [22]. To a suspension of selenium (40 mg, 0.5 mmol) in deaerated isopropanol (5 mL) was added sodium borohydride (24 mg, 0.6 mmol) in one portion at room temperature. The mixture was stirred under a nitrogen atmosphere to provide a colorless solution of NaHSe in isopropanol, which was ready to be used.

**Preparation of an aqueous solution of sodium hydrogenselenide** [22]. To a suspension of selenium (40 mg, 0.5 mmol) in deaerated distilled water (5 mL) was added sodium borohydride (40 mg, 1.0 mmol) in one portion at 5 °C. The mixture was vigorously stirred under a nitrogen atmosphere to provide a colorless aqueous solution of NaHSe, which was ready to be used.

**Synthesis of aminoacyl-*p*NAs and aminoacyl-AMCs starting from amino acid-OSu esters (procedure A).** In an ice-water bath, to a 0.05 M isopropanolic or aqueous solution of NaHSe (0.5 mmol in 10 mL), freshly prepared as described above, was added a solution of *N^α^*-protected amino acid-OSu (0.5 mmol) in THF (10 mL) by syringe. The resulting mixture was stirred at 0–5 °C for 1 h under nitrogen atmosphere to afford the corresponding *N^α^*-protected amino selenocarboxylate. Then, a solution of the azide (0.42 mmol) in THF (2 mL) was added to the above solution of amino selenocarboxylate by a syringe. The reaction was carried out at room temperature under a nitrogen atmosphere for 2 h. The organic solvents were removed under reduced pressure, and the residue was suspended in a saturated NaHCO_3_ aqueous solution followed by extraction with CH_2_Cl_2._ The combined organic phase was washed with water and brine, and dried over anhydrous Na_2_SO_4_. After removal of Na_2_SO_4_ by filtration, the filtrate was concentrated to dryness. The crude product was purified by flash column chromatography (FCC) on silica gel. The physical and spectroscopic data of all amides were consistent with their structures.

**Synthesis of aminoacyl-*p*NAs and aminoacyl-AMCs starting from amino acids by activation with isopropyl chloroformate (procedure B).** To a solution of *N^α^*-protected amino acid (0.5 mmol) and *N*-methylpiperidine (61 µL, 0.5 mmol) in THF (5 mL) was added a 1.0 M solution of isopropyl chloroformate in toluene (0.5 mL, 0.5 mmol) at –15 °C under a nitrogen atmosphere. The resulting mixture was stirred for 20 min at –15 °C. Then, the resulting mixed anhydride solution was added into the freshly prepared NaHSe (0.5 mmol) solution by cannulation over a period of 5 min. The reaction mixture was stirred for an additional 30 min below –10 °C under a nitrogen atmosphere. Then, a solution of azide (0.42 mmol) in THF was added into the above selenocarboxylate solution by cannulation. The reaction was carried out at room temperature under a nitrogen atmosphere for 2 h. The organic solvents were removed at reduced pressure, and the residue was suspended in a saturated NaHCO_3_ aqueous solution followed by extraction with CH_2_Cl_2._ The combined organic phase was washed with water and brine, and dried over anhydrous Na_2_SO_4_. After removal of Na_2_SO_4_ through filtration, the filtrate was concentrated to dryness. The crude product was purified by FCC on silica gel. Yields and physical and spectroscopic data of all amides were consistent with their structures.

## Supporting Information

Analytical data and yields for compounds **1**–**20**, among which compounds **10** and **12**–**14** are new compounds.

File 1 Analytical data and yields for compounds **1**–**20**.
