# Supplementary material for: A novel high-yield synthesis of aminoacyl p-nitroanilines and aminoacyl 7-amino-4-methylcoumarins: Important synthons for the synthesis of chromogenic/fluorogenic protease substrates
Source: Beilstein J Org Chem. 2011 Jul 27;7:1030–5. doi: 10.3762/bjoc.7.117 (PMC3167743; doi:10.3762/bjoc.7.117)
Supplement: File 1 — Analytical data and yields for compounds 1–20. [file Beilstein_J_Org_Chem-07-1030-s001.pdf]

Supporting Information

for

A novel high-yield synthesis of aminoacyl *p*-nitroanilines and aminoacyl 7-amino-4-methylcoumarins: Important synthons for the synthesis of chromogenic/fluorogenic protease substrates

Xinghua Wu[1], Yu Chen[1], Herve Aloysius[1] and Longqin Hu[*1,2,§]

[1]Department of Medicinal Chemistry, Ernest Mario School of Pharmacy, Rutgers, The State University of New Jersey, Piscataway, New Jersey 08854 and [2]The Cancer Institute of New Jersey, New Brunswick, New Jersey 08901

Email: Longqin Hu[*] - LongHu@rutgers.edu

* Corresponding author

§ Phone: 732-445-5291. Fax: 732-445-6312.

Analytical data and yields for compounds **1–20**

Synthesis of aminoacyl-*p*NAs and aminoacyl-AMCs starting from amino acid-OSu esters (procedure A).

***N*$^{\alpha}$-Benzyloxycarbonyl-L-glycyl-*p*-nitroanilide (1)**. The product (135 mg) was obtained after FCC [CH_2Cl_2 to CH_2Cl_2/EtOAc (50:50)] in 98% isolated yield. ^{1}H NMR (DMSO-d_6, 200 MHz): δ 10.57 (brs, -NH), 8.22 (d, 2H, *J* = 9.2 Hz), 7.83 (d, 2H, *J* = 9.2 Hz), 7.61 (t, -NH, *J* = 5.8 Hz), 7.36 (brs, 5H), 5.05 (s, 2H), 3.87 (d, 2H, *J* = 5.8 Hz); ^{13}C NMR (DMSO-d_6, 50 MHz): δ 169.0, 156.6, 145.0, 142.3, 137.0, 128.3, 127.8, 127.7, 125.0, 118.8, 65.6, 44.3; MS (ESI$^-$): *m*/*z* (intensity), 328.0 ([M – H]$^-$, 70%), 442.0 ([M – H]$^-$ + TFA, 100%).

***N*$^{\alpha}$-Benzyloxycarbonyl-L-tyrosyl-*p*-nitroanilide (2)**. The product (164 mg) was obtained after FCC [CH_2Cl_2 to CH_2Cl_2/EtOAc (50:50)] in 90% isolated yield. ^{1}H NMR (DMSO-d_6, 200 MHz): δ 10.69 (s, 1H, -NH), 9.20 (s, 1H, -OH), 8.22 (d, 2H, *J* = 9.2 Hz), 7.84 (d, 2H, *J* = 9.2 Hz), 7.30 (brs, 5H), 7.11 (d, 2H, *J* = 8.0 Hz), 6.65 (d, 2H, *J* = 8.2 Hz), 4.98 (s, 2H), 4.32–4.39 (m, 1H), 2.69–2.97 (m, 2H). ^{13}C NMR (DMSO-d_6, 50 MHz): δ 171.8, 155.9, 155.8, 145.0, 142.4, 136.9, 130.1, 128.3, 127.7, 127.5, 127.4, 124.9, 119.0, 114.9, 65.4, 57.5, 36.5; MS (ESI$^-$): *m*/*z* (intensity), 434.2 ([M – H]$^-$, 20%), 548.2 ([M – H]$^-$ + TFA, 100%); HRMS (ESI$^+$) *m*/*z* calcd for $C_{23}H_{22}N_3O_6$ [MH]$^+$ 436.1509, found: 436.1505.

***N*$^{\alpha}$-(9-Fluorenyloxycarbonyl)-L-threonyl-*p*-nitroanilide (3)**. The product (176 mg) was obtained after FCC (CH_2Cl_2/MeOH, 20:1) in 91% isolated yield. ^{1}H NMR (DMSO-d_6, 200 MHz): δ 10.54 (s, -NH), 8.23 (d, 2H, *J* = 9.2 Hz), 7.83−7.87 (m, 4H), 7.75 (d, 2H, *J* = 7.2 Hz), 7.23−7.41 (m, 5H), 4.95 (d, 1H, *J* = 5.8 Hz), 4.05−4.35 (m, 5H), 1.12 (d, 3H, *J* = 6.2 Hz), 1.03 (d, -OH, *J* = 6.2 Hz); ^{13}C NMR (DMSO-d_6, 50 MHz): δ 170.3, 156.2, 144.9, 143.8, 142.3, 140.7, 127.6, 127.0, 125.2, 124.9, 120.0, 119.0, 66.6, 65.8, 61.6, 46.7, 20.1; MS (ESI$^-$): *m*/*z* (intensity), 460.2 ([M – H]$^-$, 20%), 574.3 ([M – H]$^-$ + TFA, 100%).

***N*^α^-(9-Fluorenyloxycarbonyl)-L-isoleucyl-*p*-nitroanilide (4)**. The product (179 mg) was obtained after FCC (CH_2Cl_2/MeOH, 40:1) in 90% isolated yield. ^{1}H NMR (DMSO-d_6, 200 MHz): δ 10.71 (s, -NH), 8.22 (d, 2H, *J* = 8.8 Hz), 7.85–7.90 (m, 4H), 7.71–7.78 (m, 3H), 7.26–7.43 (m, 4H), 4.23–4.31 (m, 3H), 4.07 (t, 1H, *J* = 8.4 Hz), 1.80–1.88 (m, 1H), 1.40–1.56 (m, 1H), 1.16–1.26 (m, 1H), 0.81–0.89 (m, 6H); ^{13}C NMR (DMSO-d_6, 50 MHz): δ 171.8, 156.2, 144.8, 143.8, 142.3, 140.7, 127.6, 126.9, 125.2, 124.9, 120.0, 119.0, 65.7, 60.0, 46.7, 36.0, 24.5, 15.2, 10.6; MS (ESI$^-$): *m*/*z* (intensity), 508.3 ([M – H]$^-$ + 2 H_2O, 20%), 586.3 ([M – H]$^-$ + TFA, 100%); HRMS (ESI$^+$) *m*/*z* calcd for $C_{27}H_{28}N_3O_5$ [MH]$^+$ 474.2029, found: 474.2027.

***N*^α^-*t*-Butyloxycarbonyl-L-phenylalanyl-*p*-nitroanilide (5)**. The product (147 mg) was obtained after FCC (CH_2Cl_2/MeOH, 50:1) in 91% isolated yield. ^{1}H NMR ($CDCl_3$, 200 MHz): δ 9.17 (brs, -NH), 8.04 (d, 2H, *J* = 9.2 Hz), 7.49 (d, 2H, *J* = 9.2 Hz), 7.18–7.30 (m, 5H), 5.46 (d, -NH, *J* = 7.8 Hz), 4.55–4.70 (m, 1H), 3.19 (dd, 1H, *J* = 6.2 Hz, *J* = 14 Hz), 3.04 (dd, 1H, *J* = 8.0 Hz, *J* = 14 Hz), 1.39 (s, 9H); ^{13}C NMR ($CDCl_3$, 50 MHz): δ 170.7, 156.3, 143.5, 143.4, 136.0, 129.1, 128.8, 127.2, 124.7, 119.1, 81.1, 27.0, 38.1, 28.2; MS (ESI$^-$): *m*/*z* (intensity), 384.2 ([M – H]$^-$, 25%), 498.2 ([M – H]$^-$ + TFA, 100%).

***N*^α^-(9-Fluorenyloxycarbonyl)-L-methionyl-*p*-nitroanilide (6)**. The product (190 mg) was obtained after FCC (CH_2Cl_2/MeOH, 50:1) in 92% isolated yield. ^{1}H NMR (CD_3COCD_3-d_6, 200 MHz): δ 9.90 (s, -NH), 8.17 (d, 2H, *J* = 8.8 Hz), 7.88 (d, 2H, *J* = 8.8 Hz), 8.11 (d, 2H, *J* = 7.2 Hz), 7.65–7.67 (m, 2H), 7.23–7.39 (m, 4H), 6.98 (d, -NH, *J* = 7.6 Hz), 4.33–4.48 (m, 3H), 4.21 (q, 1H, *J* = 8.8 Hz), 2.04–2.20 (m, 7H); ^{13}C NMR (CD_3COCD_3-d_6, 50 MHz): δ 172.0, 157.3, 145.7, 145.0, 144.1, 142.1, 128.5, 127.9, 126.1, 125.5, 120.8, 120.2, 67.3, 56.2, 48.1, 32.5, 29.8, 15.2; MS (ESI$^-$): *m*/*z* (intensity), 604.2 ([M – H]$^-$ + TFA, 100%).

***N*^α^-(9-Fluorenyloxycarbonyl)-L-tryptophanyl-*p*-nitroanilide (7)**. The product (216 mg) was obtained after FCC (CH_2Cl_2/MeOH, 50:1) in 94% isolated yield. ^{1}H NMR (DMSO-d_6, 200

MHz): δ 10.84 (s, -NH), 10.74 (s, -NH), 8.23 (d, 2H, J = 8.8 Hz), 7.85–7.89 (m, 5H), 7.68 (m, 3H), 7.24–7.38 (m, 6H), 6.95–7.10 (m, 2H), 4.51–4.54 (m, 1H), 4.21 (m, 3H), 3.02–3.27 (m, 2H); ^{13}C NMR (DMSO-d_6, 50 MHz): δ 172.4, 156.4, 145.5, 144.2, 142.8, 141.1, 136.5, 128.0, 127.6, 127.4, 125.6, 124.3, 121.3, 120.4, 120.0, 119.6, 118.9, 118.7, 111.7, 110.1, 66.2, 56.9, 47.0, 28.0; MS (ESI$^-$): *m*/*z* (intensity), 659.3 ([M – H]$^-$ + TFA, 100%).

***N*$^{\alpha}$-(9-Fluorenyloxycarbonyl)-*N*$^{\varepsilon}$-trityl-L-histidyl-*p*-nitroanilide (8)**. The product (280 mg) was obtained after FCC (CH_2Cl_2/MeOH, 20:1) in 91% isolated yield. ^{1}H NMR ($CDCl_3$, 200 MHz): δ 10.87 (brs, 1H, -NH), 8.11 (d, 2H, J = 8.8 Hz), 7.68–7.74 (m, 4H), 7.53–7.56 (m, 3H), 7.47 (s, 1H), 7.24–7.39 (m, 12H), 7.04–7.07 (m, 7H), 6.71 (s, 1H), 4.741–4.77 (m, 1H), 4.34 (d, 2H, J = 6.6 Hz), 4.17 (t, 1H, J = 6.6 Hz), 3.10 (d, 2H, J = 6.2 Hz); ^{13}C NMR (DMSO-d_6, 50 MHz): δ 170.5, 156.2, 144.1, 143.7, 143.6, 143.2, 142.0, 141.1, 138.4, 136.3, 129.5, 128.1, 128.0, 127.6, 126.9, 125.0, 124.7, 119.9, 119.7, 119.1, 75.4, 67.2, 55.6, 47.0, 31.0; MS (ESI$^+$): *m*/*z* (intensity), 243.1 (Ph_3C^+, 100%); HRMS (ESI$^+$) *m*/*z* calcd for $C_{46}H_{38}N_5O_5$ [MH]$^+$ 740.2873, found: 740.2866.

7-(*N*$^{\alpha}$-Benzyloxycarbonyl-L-glycyl)amino-4-methylcoumarin (9). The product (141 mg) was obtained after FCC (Hexane/EtOAc, 5:1) in 92% isolated yield. ^{1}H NMR (DMSO-d_6, 200 MHz): δ 10.44 (s, -NH), 7.73 (s, 1H), 7.66 (d, 1H, J = 8.4 Hz), 7.47 (d, 1H, J = 8.4 Hz), 7.36 (brs, 5H), 6.25 (s, 1H), 5.06 (s, 2H), 3.86 (s, 2H), 2.38 (s, 3H); ^{13}C NMR (DMSO-d_6, 50 MHz): δ 168.8, 160.0, 156.6, 153.7, 153.1, 142.2, 137.0, 128.3, 127.8, 127.7, 126.0, 115.1, 115.0, 112.3, 105.6, 65.6, 44.2, 17.9; MS (ESI$^-$): *m*/*z* (intensity), 364.9 ([M – H]$^-$, 100%), 479.1 ([M – H]$^-$ + TFA, 100%).

7-(*N*$^{\alpha}$-Benzyloxycarbonyl-L-tyrosyl)amino-4-methylcoumarin (10). The product (170 mg) was obtained after FCC (Hexane/EtOAc, 4:1) in 86% isolated yield. ^{1}H NMR (DMSO-d_6, 200 MHz): δ 10.51 (s, 1H, -NH), 9.21 (s, 1H, -OH), 7.76 (s, 1H), 7.69 (d, 1H, J = 8.8 Hz), 7.47

(d, 1H, J = 8.8 Hz), 7.31 (brs, 5H), 7.12 (d, 2H, J = 8.0 Hz), 6.67 (d, 2H, J = 8.6 Hz), 6.24 (s, 1H), 4.98 (s, 2H), 4.37 (dd, 1H, J = 4.0 Hz, J = 8.0 Hz), 2.71–2.97 (m, 2H), 2.37 (s, 3H); ^{13}C NMR (DMSO-d_6, 50 MHz): δ 171.5, 160.0, 156.0, 153.6, 153.0, 142.1, 136.9, 130.2, 128.3, 127.7, 127.6, 125.8, 115.3, 115.1, 114.9, 112.3, 105.8, 65.4, 27.4, 36.7, 17.9. MS (ESI$^-$): *m*/*z* (intensity), 471.0 ([M – H]$^-$, 70%), 585.1 ([M – H]$^-$ + TFA, 100%); HRMS (ESI$^+$) *m*/*z* calcd for $C_{27}H_{25}N_2O_6$ [MH]$^+$ 473.1713, found: 473.1697.

7-(N^{α}-*t*-Butyloxycarbonyl-L-phenylalanyl)amino-4-methylcoumarin (11). The product (152 mg) was obtained after FCC (Hexane/EtOAc, 1:1) in 86% isolated yield. ^{1}H NMR ($CDCl_3$, 200 MHz): δ 9.11 (brs, -NH), 7.65 (s, 1H), 7.37 (d, 1H, J = 8.4 Hz), 7.21 (brs, 5H), 7.07 (m, 1H), 6.08 (s, 1H), 5.40 (d, -NH, J = 7.6 Hz), 4.66 (m, 1H), 3.17 (dd, 1H, J = 6.2 Hz, J = 14 Hz), 3.01 (dd, 1H, J = 8.4 Hz, J = 14 Hz), 2.33 (s, 3H), 1.39 (s, 9H); ^{13}C NMR ($CDCl_3$, 50 MHz): δ 170.6, 160.9, 156.2, 153.9, 152.4, 141.0, 136.1, 129.1, 128.7, 127.0, 124.9, 115.8, 115.5, 113.1, 107.2, 80.8, 56.7, 38.3, 28.3, 18.3; MS (ESI$^-$): *m*/*z* (intensity), 421.2 ([M – H]$^-$, 30%), 535.2 ([M – H]$^-$ + TFA, 100%).

7-(N^{α}-(9-Fluorenyloxycarbonyl)-L-methionyl)amino-4-methylcoumarin (12). The product (182 mg) was obtained after FCC (Hexane/EtOAc, 2:1) in 82% isolated yield. ^{1}H NMR (DMSO-d_6, 200 MHz): δ 10.5 (brs, -NH), 7.68–7.89 (m, 7H), 7.28–7.53 (m, 4H and -NH), 6.25 (s, 1H), 4.20–4.30 (m, 4H), 2.50–2.55 (m, 2H), 2.38 (s, 3H), 2.06 (s, 3H), 1.97–2.04 (m, 2H); ^{13}C NMR (DMSO-d_6, 50 MHz): δ 171.8, 160.4, 156.6, 154.1, 153.4, 144.3, 142.6, 141.2, 128.1, 127.5, 126.3, 125.7, 120.5, 115.8, 115.6, 112.8, 106.3, 66.2, 55.3, 47.1, 31.8, 30.2, 18.4, 15.1; MS (ESI$^-$): *m*/*z* (intensity), 641.3 ([M – H]$^-$ + TFA, 100%); HRMS (ESI$^+$) *m*/*z* calcd for $C_{30}H_{29}N_2O_5S$ [MH]$^+$ 529.1797, found: 529.1783.

7-(N^{α}-(9-Fluorenyloxycarbonyl)-L-tryptophanyl)amino-4-methylcoumarin (13). The product (200 mg) was obtained after FCC (Hexane/CH_2Cl_2/MeOH, 10:10:1) in 82% isolated yield. ^{1}H

NMR (DMSO-d_6, 200 MHz): δ 10.85 (brs, -NH), 10.58 (brs, -NH), 7.82–7.87 (m, 4H), 7.65–7.71 (m, 4H), 7.25–7.53 (m, 6H and -NH), 6.96–7.11 (m, 2H), 6.25 (s, 1H), 4.53 (m, 1H), 4.21 (brs, 3H), 3.10–3.27 (m, 2H), 2.38 (s, 3H); ^{13}C NMR (DMSO-d_6, 50 MHz): δ 171.7, 160.0, 155.9, 153.6, 152.9, 143.7, 142.2, 140.6, 136.1, 127.5, 127.2, 127.0, 125.7, 125.2, 123.9, 120.9, 120.0, 118.5, 118.2, 115.4, 115.1, 112.3, 111.3, 109.7, 105.9, 65.7, 56.4, 46.6, 27.7, 17.9; MS (ESI$^-$): *m/z* (intensity), 696.2 ([M – H]$^-$ + TFA, 100%); HRMS (ESI$^+$) *m/z* calcd for $C_{36}H_{30}N_3O_5$ [MH]$^+$ 584.2185, found: 584.2172.

7-(N^{α}-(9-Fluorenyloxycarbonyl)-N^{ε}-trityl-L-histidyl)amino-4-methylcoumarin (14). The product (268 mg) was obtained after FCC (Hexane/EtOAc, 1:1) in 82% isolated yield. ^{1}H NMR ($CDCl_3$, 200 MHz): δ 10.57 (brs, 1H, -NH), 7.71 (d, 2H, *J* = 7.4 Hz), 7.67 (s, 1H), 7.56 (d, 2H, *J* = 6.2 Hz), 7.46 (s, 1H), 7.23–7.37 (m, 14H), 7.03–7.06 (m, 6H), 6.91 (d, 1H, *J* = 6.6 Hz), 6.68 (s, 1H), 6.12 (s,1H), 4.74 (q, 1H, *J* = 6.2 Hz), 4.33 (d, 2H, *J* = 6.6 Hz), 4.17 (t, 1H, *J* = 6.6 Hz), 3.10 (brs, 2H), 2.34 (s,3H). ^{13}C NMR ($CDCl_3$, 50 MHz): δ 170.2, 160.8, 156.3, 154.0, 152.0, 143.7, 143.6, 142.0, 141.5, 141.1, 138.3, 136.3, 129.5, 127.9, 127.5, 126.9, 125.0, 124.8, 119.8, 119.7, 115.6, 115.5, 113.0, 106.9, 75.3, 67.2, 55.7, 46.9, 30.9, 18.3. MS (ESI$^+$): *m/z* (intensity), 243.1 (Ph_3C^+, 100%); HRMS (ESI$^+$) *m/z* calcd for $C_{50}H_{41}N_4O_5$ [MH]$^+$ 777.3077, found: 777.3057.

Synthesis of aminoacyl-*p*NAs and aminoacyl-AMCs starting from amino acids via activation with isopropyl chloroformate (procedure B).

N^{α}-*tert*-Butyloxycarbonyl-L-glutaminyl-*p*-nitroanilide (15). The product (140 mg) was obtained after FCC (Hexane/CH_2Cl_2/MeOH, 10:10:1) in 91% isolated yield. ^{1}H NMR (DMSO-d_6, 200 MHz): δ 10.60 (s, -NH), 8.22 (d, 2H, *J* = 9.2 Hz), 7.851 (d, 2H, *J* = 9.2 Hz), 7.29 (s, -NH), 7.18(d, -NH, *J* = 6.8 Hz), 6.79 (s, -NH), 4.05 (m, 1H), 2.11–2.22 (m, 2H), 1.77–1.88 (m, 2H), 1.36 (s, 9H); ^{13}C NMR (DMSO-d_6, 50 MHz): δ 173.4, 172.0, 155.4, 145.0, 142.2,

124.8, 118.9, 78.2, 55.1, 31.4, 28.1, 27.1; MS (ESI$^-$): *m*/*z* (intensity), 365.1 ([M – H]$^-$, 20%), 479.2 ([M – H]$^-$ + TFA, 100%); HRMS (ESI$^+$) *m*/*z* calcd for $C_{16}H_{23}N_4O_6$ [MH]$^+$ 367.1618, found: 367.1612.

***N*$^{\alpha}$-*tert*-Butyloxycarbonyl-L-arginyl-*p*-nitroanilide (16)**. The product (134 mg) was obtained after FCC (CH_2Cl_2/MeOH, 20:1) in 81% isolated yield. ^{1}H NMR (CD_3OD, 200 MHz): δ 8.28 (d, 2H, *J* = 9.0 Hz), 7.95 (d, 2H, *J* = 9.0 Hz), 4.32 (m, 1H), 1.76–1.96 (m, 4H), 1.49 (s, 9H), 1.37 (t, 2H, *J* = 7.4 Hz); ^{13}C NMR (CD_3OD, 50 MHz): δ 169.6, 154.5, 141.7, 140.6, 121.5, 116.5, 76.8, 52.3, 37.9, 26.3, 24.5, 22.3; MS (ESI$^+$): *m*/*z* (intensity), 395.3 ([M + H]$^+$, 100%).

***N*$^{\alpha}$-Benzyloxycarbonyl-L-seryl-*p*-nitroanilide (17)**. The product (130 mg) was obtained after FCC (CH_2Cl_2/MeOH, 20:1) in 86% isolated yield. ^{1}H NMR (DMSO-*d*$_6$, 200 MHz): δ 10.65 (s, -NH), 8.22 (d, 2H, *J* = 9.2 Hz), 7.88 (d, 2H, *J* = 9.2 Hz), 7.44 (m, 6H), 5.05 (s, 2H), 4.25–4.35 (m, 1H), 3.60–3.70 (m, 2H); ^{13}C NMR (DMSO-*d*$_6$, 50 MHz): δ 170.4,156.0, 145.1, 144.9, 142.4, 136.9, 128.3, 127.8, 124.9, 119.0, 65.7, 61.6, 58.0; MS (ESI$^-$): *m*/*z* (intensity), 358.1 ([M – H]$^-$, 30%), 472.2 ([M – H]$^-$ +TFA, 100%).

***N*$^{\alpha}$-*tert*-Butyloxycarbonyl-L-seryl-L-phenylalanyl-*p*-nitroanilide (18)**. The product (176 mg) was obtained after FCC (CH_2Cl_2/MeOH, 20:1) in 89% isolated yield. ^{1}H NMR ($CDCl_3$, 200 MHz): δ 9.08 (s, -NH), 8.05 (d, 2H, *J* = 9.2 Hz), 7.768 (d, 2H, *J* = 8.8 Hz), 7.12–7.28 (m, 5H), 5.61 (d, -NH, *J* = 4.4 Hz), 4.87 (q, 1H, *J* = 6.2 Hz), 4.13 (t, 1H, *J* = 6.2 Hz), 3.64–3.92 (m, 2H), 3.19–3.4 (m, 2H), 1.33(s, 9H). ^{13}C NMR ($CDCl_3$, 50 MHz): δ 171.3, 169.9, 156.3, 143.6, 143.5, 135.7, 129.1, 128.9, 127.4, 124.7, 119.5, 81.2, 62.4, 56.7, 55.0, 37.4, 28.2; MS (ESI$^-$): *m*/*z* (intensity), 585.3 ([M – H]$^-$ + TFA, 100%); HRMS (ESI$^+$) *m*/*z* calcd for $C_{23}H_{29}N_4O_7$ [MH]$^+$ 473.2036, found: 473.2024.

7-(*N*$^{\alpha}$-*tert*-Butyloxycarbonyl-L-glutaminyl)amino-4-methylcoumarin (19). The product (142 mg) was obtained FCC (CH_2Cl_2/MeOH, 4:1) in 84% isolated yield. ^{1}H NMR (CD_3OD,

400 MHz): δ 7.70 (d, 1H, J = 2.0 Hz), 7.60 (d, 1H, J = 8.7 Hz), 7.39 (d, 1H, J = 8.2 Hz), 6.13 (s, 1H), 4.12 (s, 1H), 2.35 (s, 3H), 2.28 (t, 2H, J = 7.5 Hz), 2.02 (m, 1H), 1.87 (m, 1H), 1.36 (s, 9H); ^{13}C NMR ($CDCl_3$, 400 MHz): δ 177.8, 173.4, 163.2, 157.9, 155.4, 155.2, 149.9, 143.4, 126.7, 117.3, 117.2, 113.7, 108.1, 81.0, 56.5, 32.5, 29.1, 28.8, 18.5; MS (ESI$^+$): *m*/*z* (intensity), 403.7 ([M + H]$^+$, 12%), 706.7 ([2M + H-Boc]$^+$, 100%); HRMS (ESI$^+$) *m*/*z* calcd for $C_{20}H_{26}N_3O_6$ [MH]$^+$ 404.1822, found: 404.1807.

7-(N^{α}-*tert*-Butyloxycarbonyl-L-arginyl)amino-4-methylcoumarin (20). The product (149 mg) was obtained FCC (CH_2Cl_2/MeOH, 4:1) in 82% isolated yield. ^{1}H NMR (CD_3OD, 400 MHz): δ 7.84 (d, 1H, J = 2.0 Hz), 7.74 (d, 1H, J = 8.7 Hz), 7.51 (d, 1H, J = 8.5 Hz), 6.27 (s, 1H), 4.25 (s, 1H), 3.25 (t, 2H, J = 7.1 Hz), 2.48 (s, 3H), 1.90 (q, 2H, J = 6.3 Hz, J = 6.9 Hz), 1.74 (m, 2H), 1.48 (s, 9H); ^{13}C NMR ($CDCl_3$, 400 MHz): δ 177.9, 173.6, 163.2, 158.7, 158.0, 155.4, 155.2, 143.3, 126.8, 117.4, 117.2, 113.8, 108.1, 81.0, 56.3, 42.0, 30.6, 28.7, 26.5, 18.5; MS (ESI$^+$): *m*/*z* (intensity), 432.1 ([M + H]$^+$, 100%).

7-(N^{α}-*t*-Butyloxycarbonyl-L-phenylalanyl)amino-4-methylcoumarin (11). The product (154 mg) was obtained FCC (CH_2Cl_2/MeOH, 4:1) in 87% isolated yield. Spectroscopic data were the same for the compound obtained using the two procedures A and B.
